# DTI-ALPS: An MR biomarker for motor dysfunction in patients with subacute ischemic stroke

**DOI:** 10.3389/fnins.2023.1132393

**Published:** 2023-03-31

**Authors:** Yue Qin, Xin Li, Yanqiang Qiao, Huili Zou, Yifan Qian, Xiaoshi Li, Yinhu Zhu, Wenli Huo, Lei Wang, Ming Zhang

**Affiliations:** ^1^Department of Medical Imaging, The First Affiliated Hospital of Xi'an Jiaotong University, Xi'an, China; ^2^Department of Radiology, Xi'an Daxing Hospital, Xi'an, China; ^3^Department of Rehabilitation Medicine, Xi'an Daxing Hospital, Xi'an, China

**Keywords:** glymphatic system, ischemic stroke, analysis along perivascular space, corticospinal tract, motor dysfunction

## Abstract

**Purpose:**

Brain glymphatic dysfunction is involved in the pathologic process of acute ischemic stroke (IS). The relationship between brain glymphatic activity and dysfunction in subacute IS has not been fully elucidated. Diffusion tensor image analysis along the perivascular space (DTI-ALPS) index was used in this study to explore whether glymphatic activity was related to motor dysfunction in subacute IS patients.

**Methods:**

Twenty-six subacute IS patients with a single lesion in the left subcortical region and 32 healthy controls (HCs) were recruited in this study. The DTI-ALPS index and DTI metrics (fractional anisotropy, FA, and mean diffusivity, MD) were compared within and between groups. Spearman's and Pearson's partial correlation analyses were performed to analyze the relationships of the DTI-ALPS index with Fugl-Meyer assessment (FMA) scores and with corticospinal tract (CST) integrity in the IS group, respectively.

**Results:**

Six IS patients and two HCs were excluded. The left DTI-ALPS index of the IS group was significantly lower than that of the HC group (*t* = −3.02, *p* = 0.004). In the IS group, a positive correlation between the left DTI-ALPS index and the simple Fugl-Meyer motor function score (ρ = 0.52, *p* = 0.019) and a significant negative correlation between the left DTI-ALPS index and the FA (*R* = −0.55, *p* = 0.023) and MD (*R* = −0.48, *p* = 0.032) values of the right CST were found.

**Conclusions:**

Glymphatic dysfunction is involved in subacute IS. DTI-ALPS could be a potential magnetic resonance (MR) biomarker of motor dysfunction in subacute IS patients. These findings contribute to a better understanding of the pathophysiological mechanisms of IS and provide a new target for alternative treatments for IS.

## 1. Introduction

Ischemic stroke (IS) remains one of the leading causes of disability and death worldwide (Campbell et al., 2019; Campbell and Khatri, 2020; Fukuta et al., 2022). Approximately 69.6% of stroke incidents in China are IS, similar to the global average (Wang W. et al., 2017; Feigin et al., 2018). The most common symptom associated with IS sensorimotor dysfunction (Langhorne et al., 2009; Alawieh et al., 2018), which can recover spontaneously within 3 months (Kwakkel et al., 2006; van der Vliet et al., 2020). A previous study has pointed out that, throughout the recovery process, early subacute IS is a critical period for neuroplasticity and recovery (Bernhardt et al., 2017).

Previous research demonstrated that brain lymphatic activity not only contributed to the pathologic process of IS but also influenced its recovery. The glymphatic system is the perivascular network used for the exchange between cerebrospinal fluid (CSF) and interstitial fluid (ISF) in the brain (Iliff et al., 2012; Klostranec et al., 2021). It consists of three main structures (Iliff et al., 2012; Benveniste et al., 2019): para-arterial CSF influx channels, para-venous ISF efflux channels, and astrocyte exchange channels connecting the two channels (aquaporin-4, AQP4). Brain glymphatic dysfunction is involved in the pathologic process of acute IS (Iliff et al., 2014; Chen S. et al., 2021). We proposed that pathological changes in subacute IS were associated with brain glymphatic dysfunction in subacute IS. The brain glymphatic system is involved in the clearance of brain metabolic waste (Choi et al., 2021). However, the brain glymphatic pathway attenuates brain edema by clearing cellular debris from ISF and promotes central nervous system (CNS) recovery after IS (Benveniste et al., 2019; Li et al., 2019; Zhou et al., 2021). Studies used variant animal models of cerebral ischemia to examine ischemia-induced functional changes at different time points and the role of AQP4 in brain edema after IS and found that the inhibition of AQP4 reduced cerebral edema and improved motor recovery and long-term prognosis (Hirt et al., 2017; Liu et al., 2017). Further research is needed to confirm whether glymphatic dysfunction in humans is related to motor dysfunction and altered white matter microstructure in subacute IS in order to understand the role of the glymphatic system in the pathophysiology of IS.

The corticospinal tract (CST) is the primary descending motor pathway carrying movement-related information and has been widely studied after stroke (Liu et al., 2020), with the main research directions being the correlation between CST injury and motor dysfunction and the prediction of motor function recovery using CST integrity after stroke (Lim et al., 2020; Hayward et al., 2022). Diffusion tensor image (DTI) metrics are currently the most common indices depicting the microstructural integrity of white matter. Among them, fractional anisotropy (FA) represents axonal alterations (Tavazzi et al., 2022), and mean diffusivity (MD) is associated with cerebral edema (Chormai et al., 2022). Previous studies showed that DTI metrics are the reliable quantitative metrics of CST that correlate with motor function outcomes and Fugl-Meyer assessment (FMA) scores in stroke rehabilitation (Haque et al., 2021; Lee et al., 2021). Although DTI metrics play an important role in describing the anatomical and pathological changes caused by IS, the lack of biological specificity and interpretation of pathophysiological disease information (for example, the mechanism of cerebral edema after IS) limit its clinical applications (Ji et al., 2021; Kamagata et al., 2021; Andica et al., 2022). Recently, a study on an epileptic seizure exploring differences in white matter integrity and glymphatic function indicated that the impairment of the glymphatic system may precede white matter microstructure in the early stage of epilepsy and implicated the potential role of measuring brain lymphatic activity in the expression and comprehensive understanding of early pathological changes of brain disorders (Salimeen et al., 2021). Therefore, quantitative measurement of changes in stroke-related lymphatic activity in CST may help explain the mechanism underlying motor disruption in IS patients and provide possible medical interference of stroke for IS patients with motor dysfunction.

Diffusion tensor image analysis along the perivascular space (DTI-ALPS) is a non-invasive method for evaluating glymphatic system function in individual subjects based on diffusion tension imaging (Taoka et al., 2017). DTI-ALPS has been demonstrated to produce results within minutes with good stability and intra-observer consistency (Si et al., 2022) and can be used as an alternative to DTI for wider use in clinical practice (Taoka et al., 2022a). Using both glymphatic magnetic resonance imaging (MRI) and DTI-ALPS methods, Zhang et al. (2021) measured and compared glymphatic clearance function and found a significant correlation between the DTI-ALPS index and the brain glymphatic clearance rate calculated by classical glymphatic MRI, indicating that DTI-ALPS might represent the precise function of brain glymphatic clearance (Siow et al., 2022). The DTI-ALPS index has been used to assess variations in the glymphatic system in several diseases, such as Alzheimer's disease, type 2 diabetes mellitus, idiopathic normal pressure hydrocephalus, Parkinson's disease, cancer pain, and other diseases (Taoka et al., 2017; Yang et al., 2020; Bae et al., 2021; Heo et al., 2021; Ma et al., 2021; Okada et al., 2021; Toh and Siow, 2021a; Wang et al., 2022). Zhang et al. (2022) used the DTI-ALPS method in patients with hemorrhagic stroke and found the impairment of the ipsilateral glymphatic system function on the lesion side. Another study used the same method to investigate glymphatic system function in patients with ischemic stroke (Toh and Siow, 2021b) and found similar results of glymphatic system dysfunction. However, these two studies did not address the relationship between glymphatic system dysfunction and stroke-related clinical symptoms, which requires further research.

In this study, we used the DTI-ALPS method to investigate glymphatic activity and white matter integrity of CST in subacute IS patients with motor dysfunction. We assumed that glymphatic activity was impaired after subacute IS, which might be related to motor dysfunction and changes in white matter microstructure.

## 2. Materials and methods

### 2.1. Participants and clinical information

**Figure 1:**
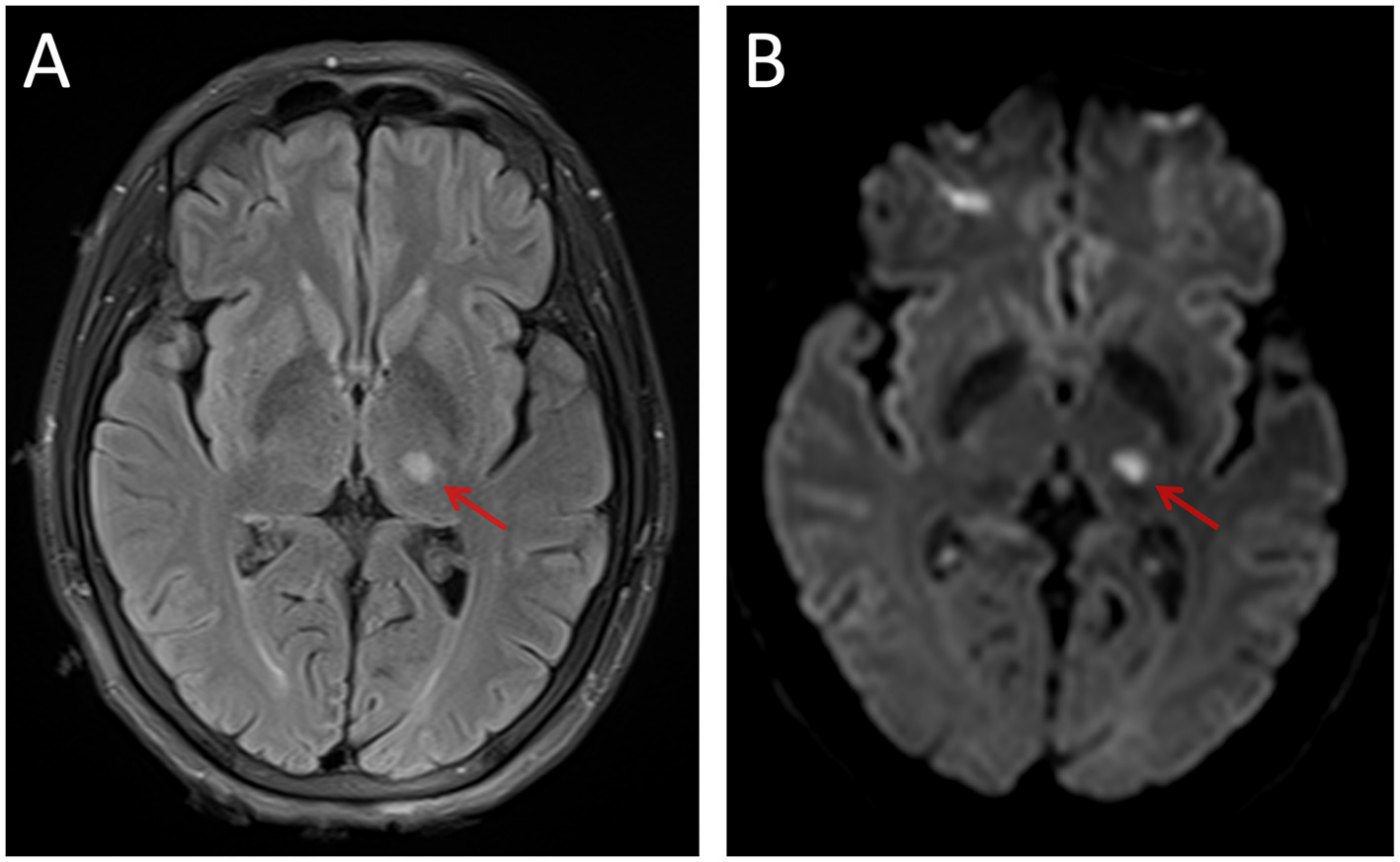
An example of the left thalamic infarct on FLAIR **(A)** and DWI **(B)**. FLAIR, fluid-attenuated inversion recovery; DWI, diffusion-weighted imaging.

From November 2020 to December 2021, 26 IS and 32 healthy controls (HCs) were recruited for this cross-sectional study. The inclusion criteria for patients with IS were as follows: (1) age being 18 years or older; (2) a single lesion in the left subcortical regions; (3) magnetic resonance (MR) images collected 7–40 days after stroke onset (Kang et al., 2012); (4) the modified Fazekas scale for white matter hyperintensities ≤2 (Fazekas et al., 1987); and (5) right-handed before stroke onset. The exclusion criteria were as follows: (1) the T2-weighted fluid-attenuated inversion recovery (FLAIR) sequence showed an ischemic lesion (Figure 1) involving regions of interest (ROI) at the level of the lateral ventricle (the largest slice of projection and association fibers shown simultaneously); (2) recurrent stroke defined by clinical history and MRI evaluation; (3) a history of major neuropsychiatric disorders (i.e., Alzheimer's disease, Parkinson's disease, schizophrenia, and epilepsy); (4) contraindications to MRI (metallic foreign body, electronic implants, or shunt pumps in the brain and body); and (5) participation in drug clinical trials. The human study protocol was approved by the medical research ethics committee of Xi'an Daxing Hospital (No. Dxll2020-153), and informed consent was obtained from all participants prior to examination.

The FMA (Busk et al., 2021) was used to evaluate motor (range: 0–100 score) and sensation (range: 0–24 score) function (Sullivan et al., 2011) in stroke patients. All assessments were completed the day before the MRI examination. FMA was performed on a one-to-one basis with the patient by an occupational therapist (Gladstone et al., 2002).

### 2.2. MR image acquisition

Image data from all participants were acquired using a 3.0T MRI scanner (MAGNETOM Prisma, Siemens Healthineers, Germany) with a 64-channel head/neck coil. Participants were placed in a supine position, and the coil was filled with a sponge pad to keep the head stationary during the examination. Conventional MR images (including T2-FLAIR images) were acquired to identify stroke lesions and other brain abnormalities. Diffusion spectrum imaging (DSI) scans were obtained with an echo planar imaging (EPI) sequence, and multiple *b*-values (repetition time = 3,300 ms, echo time = 73 ms, field of view = 220 mm × 220 mm, matrix = 110 × 110, slice thickness = 2 mm, number of slices = 60, and in-plane resolution = 2 mm) were performed. Using a multiband sequence (Simultaneous Multi-Slice = 2, GRAPPA = 2), a total of 129 DWI volumes were acquired, with 18 different *b*-values ranging from *b* = 0 to *b* = 3,000 s/mm^2^ and 128 different diffusion encoding orientations (the *b* table of one patient's DSI data is provided in Supplementary Table S1).

### 2.3. DSI processing

**Figure 2:**
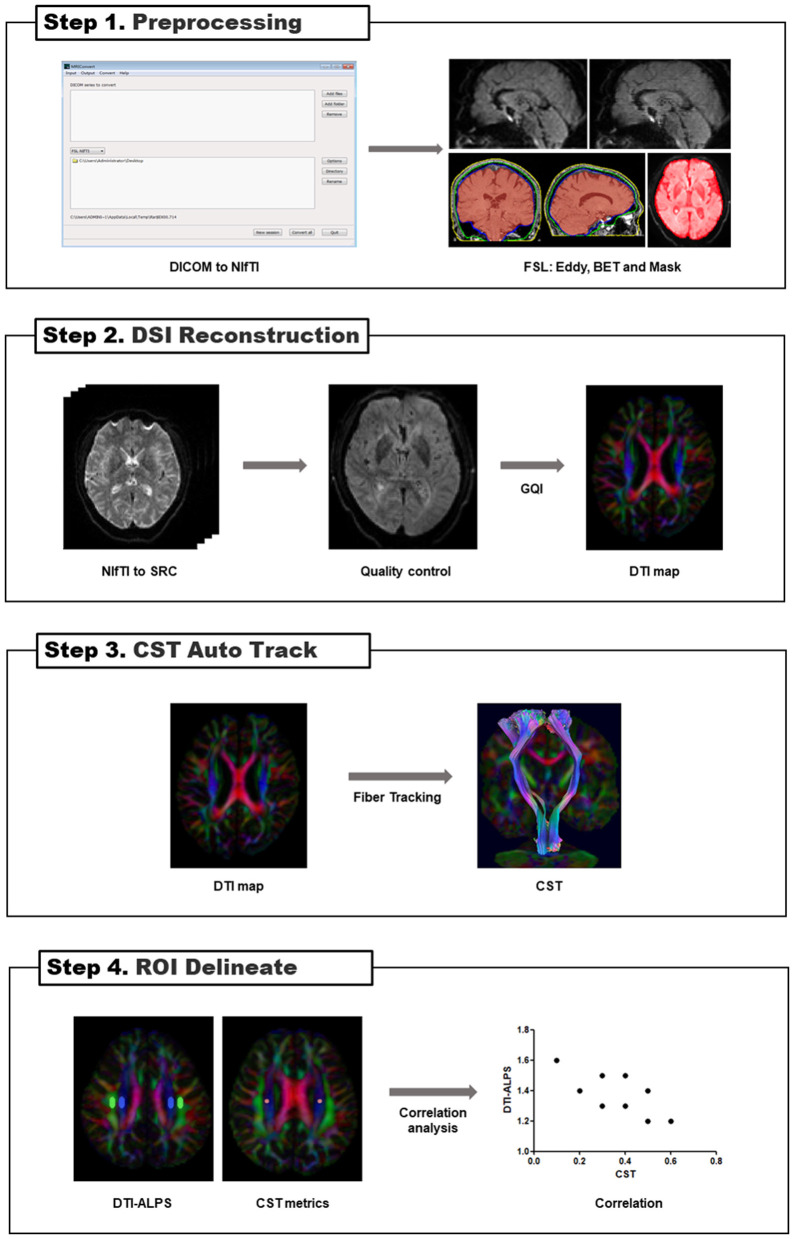
Flowcharts for DSI image processing. BET, brain extraction tool; GQI, generalized q-sampling imaging; DSI, diffusion spectrum images; DTI, diffusion tensor image; CST, corticospinal tract; DTI-ALPS, diffusion tensor image analysis along the perivascular space; ROI, regions of interest.

Diffusion spectrum images were processed using the DSI Studio software (version chen “
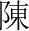
” build 13 January 2022, http://dsi-studio.labsolver.org). The FSL's (version 6.0.5.3, https://fsl.fmrib.ox.ac.uk/fsl/fslwiki) (Smith, 2002) eddy was used for eddy current and motion correction. The brain extraction tool (BET) was used to remove the whole brain's scalp and skull and generate masks. The generalized q-sampling imaging (GQI) (Yeh et al., 2010) with a diffusion sampling length ratio of 1.25 was used to reconstruct an individual space to generate a color-coded FA map. To calculate the diffusivity of water molecules in the perivascular space in axial slices, DSI volumes with a *b*-value of no more than 1,150 s/mm^2^ were used to form the DTI model. Automated tractography methods were used to reconstruct and visualize bilateral CSTs. FA, MD, and water diffusivity along the *x, y*, and *z* axes were calculated. Finally, FLAIR images were imported into the DSI studio and recorded in the DSI space to observe lesions (refer to Figure 2 and Supplementary Table S1 for the detailed process).

### 2.4. Regions of interest

**Figure 3:**
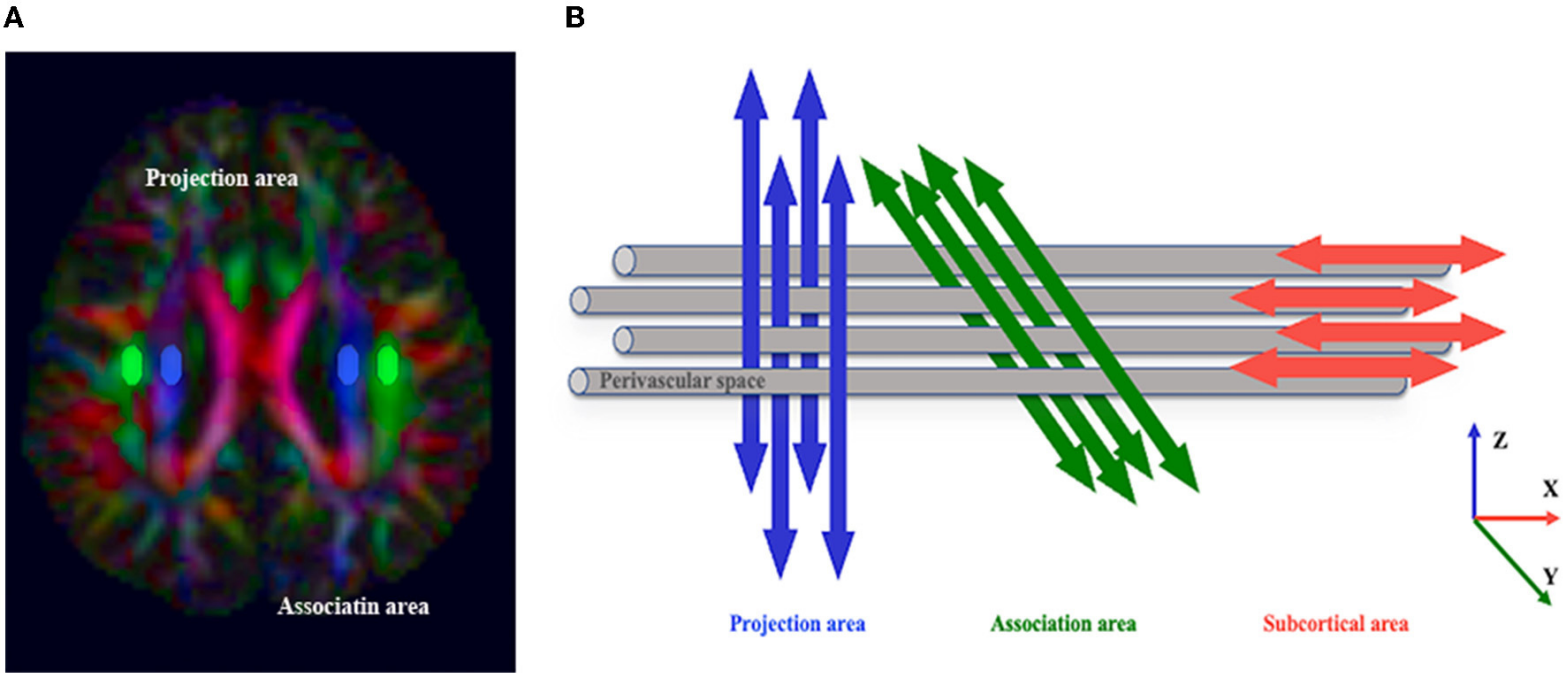
The concept of the diffusion tensor image analysis along the perivascular space (DTI-ALPS) method. **(A)** The DTI color map shows the direction of the projection fibers (*z*-axis, blue), association fibers (*y*-axis, green), and subcortical fibers (*x*-axis, red). Two regions of interest (ROIs) are placed to measure the diffusivities of the projection (projection area) and association (association area) fibers. **(B)** The schematic diagram indicates the relationship between the direction of the perivascular space (gray cylinder) and the direction of the fibers. Note that the direction of the perivascular space is perpendicular to the projection and association fibers (Taoka et al., 2017).

Based on measurement satisfaction, ROIs (volume size = 12 mm × 4 mm × 2 mm) were placed in the projection and association fiber regions of the bilateral hemisphere on the color-coded FA map in the horizontal plane of the lateral ventricle body (Taoka et al., 2017) to calculate DTI-ALPS (Figure 3). The ROIs (volume size = 4 mm × 4 mm × 2 mm) of the CST were placed in the same section. We used FLAIR images to avoid placing ROIs over visibly damaged tissue (infarct foci or white matter hyperintensity). If the lesion involved the ROI regions, the subject was excluded. To investigate whether commissural fibers that travel in the left and right directions affect the ALPS index, we placed the ROIs (volume size = 4 mm × 4 mm × 2 mm) of the corpus callosum (CC) on the same level as the bilateral hemisphere's projection and association fiber regions on the color-coded FA map, respectively (Supplementary Figure S2).

### 2.5. Calculation of the DTI-ALPS index

The DTI-ALPS index was proposed by Taoka et al. (2017), which was calculated as the ratio between the MD in the area of projection fibers (*D*_xx − proj_) and association fibers (*D*_xx − assoc_) on the *x*-axis and that of the projection fibers (*D*_yy − proj_) on the *y*-axis and the association fibers (*D*_zz − assoc_) on the *z*-axis as follows:


$$\begin{array}{l} \begin{array}{c} DTI-ALPS\;index=\frac{mean\;\left(Dxx-proj,\;Dxx-assoc\right)}{mean\;\left(Dyy-proj,\;Dzz-assoc\right)\;}\#\left(1\right) \end{array} \end{array}$$

The DTI-ALPS index was calculated for each subject to assess the activity of the glymphatic system in the native space.

### 2.6. Statistics

R statistics software (https://cran.r-project.org/, version 4.0) was used to perform demographic data analysis, intergroup comparison, and correlation analysis. All continuous data were reported as mean ± SD.

Two-sample *t*-test or Pearson's χ^2^ test was used to compare the differences between the IS and HC groups, including the demographic data and DTI-ALPS indices.

A paired *t*-test was used to study whether there was a lateralization effect on DTI-ALPS and CST metrics within each group.

We used Spearman's correlation to analyze the relationship between bilateral DTI-ALPS index and FMA scores (simple Fugl-Meyer motor function score and Fugl-Meyer sensory score) in the IS group. Controlling for age and time since stroke onset, the correlation of the DTI-ALPS index with CST metrics was assessed using Pearson's partial correlation analysis.

The relationship between the left ALPS and the left FA of CC and the right ALPS and the right FA of CC in the two groups was analyzed using Pearson's partial correlation analysis. In addition, the relationship between bilateral ALPS and the mean FA of bilateral CC was also analyzed.

If a *p*-value was < 0.05, the results were considered statistically significant after multiple comparison corrections.

## 3. Results

### 3.1. Demographics and clinical information

Initially, 6 of the 26 subcortical IS patients were excluded due to ischemic lesions involving ROI regions (*n* = 5) or the modified Fazekas scale for white matter hyperintensities >2 (*n* = 1), and two HC participants were excluded due to poor image quality. Finally, 20 IS (16 men; age range: 33–83 years; mean age: 59.2 ± 12.1 years) and 30 HCs (20 men; age range: 42–66; mean age: 54.6 ± 7.4 years) were included. The two groups were matched in terms of age (*t* = 1.50, *p* = 0.146) and gender (χ^2^ = 0.50, *p* = 0.479). The demographic and clinical characteristics of participants are summarized in Table 1.

**Table 1:** Demographic and clinical characteristics of 50 participants^a^.

	**Stroke patients**	**Healthy controls**	**χ^2^/*t*^b^**	***p*** **-value**
Number of cases	20	30	–	–
Mean age ± SD (year)	59.2 ± 12.1	54.6 ± 7.4	1.50	0.146
Men (%)	16 (80%)	20 (67%)	0.50	0.479
Time after stroke (day)^c^	9 (7–40)	–	–	–
Lesion location	Left hemisphere	–	–	–
Infarct volume (mm^3^)	216–6,928	–	–	–
Simple FM motor function score^c^	88.5 (14–100)	–	–	–
FM sensory score^c^	22 (11–24)	–	–	–
Education (years)	8.5 ± 1.8	8.2 ± 1.6	0.45	0.656
**Stroke risk factors**				
Diabetes	5 (25%)	3 (10%)	–	–
Hypertension	17 (85%)	21 (70%)	–	–
Coronary artery disease	1 (5%)	0	–	–
Current smoker	15 (75%)	18 (60%)	–	–
Carotid artery disease	19 (95%)	15 (50%)	–	–
Atrial fibrillation	2 (10%)	0	–	–

a Data are presented as the number of patients (%) or mean ± standard deviation (SD).

b Data are calculated by student's t-test or Pearson's χ^2^ test.

c Data are presented as median (range).

SD, standard deviation; FM, Fugl-Meyer.

### 3.2. Comparison of the DTI-ALPS index within and between the two groups

In the IS group, the DTI-ALPS index was 1.424 ± 0.132 (left side) and 1.381 ± 0.172 (right side); in the HC group, the index was 1.565 ± 0.197 (left side) and 1.454 ± 0.107 (right side) (refer to Supplementary Table S2).

The left DTI-ALPS index in the IS group was significantly lower than the same side in the HC group (*t* = −3.02, *p* = 0.004), but the right DTI-ALPS difference between the groups was not significant (*t* = −1.87, *p* = 0.067).

**Figure 4:**
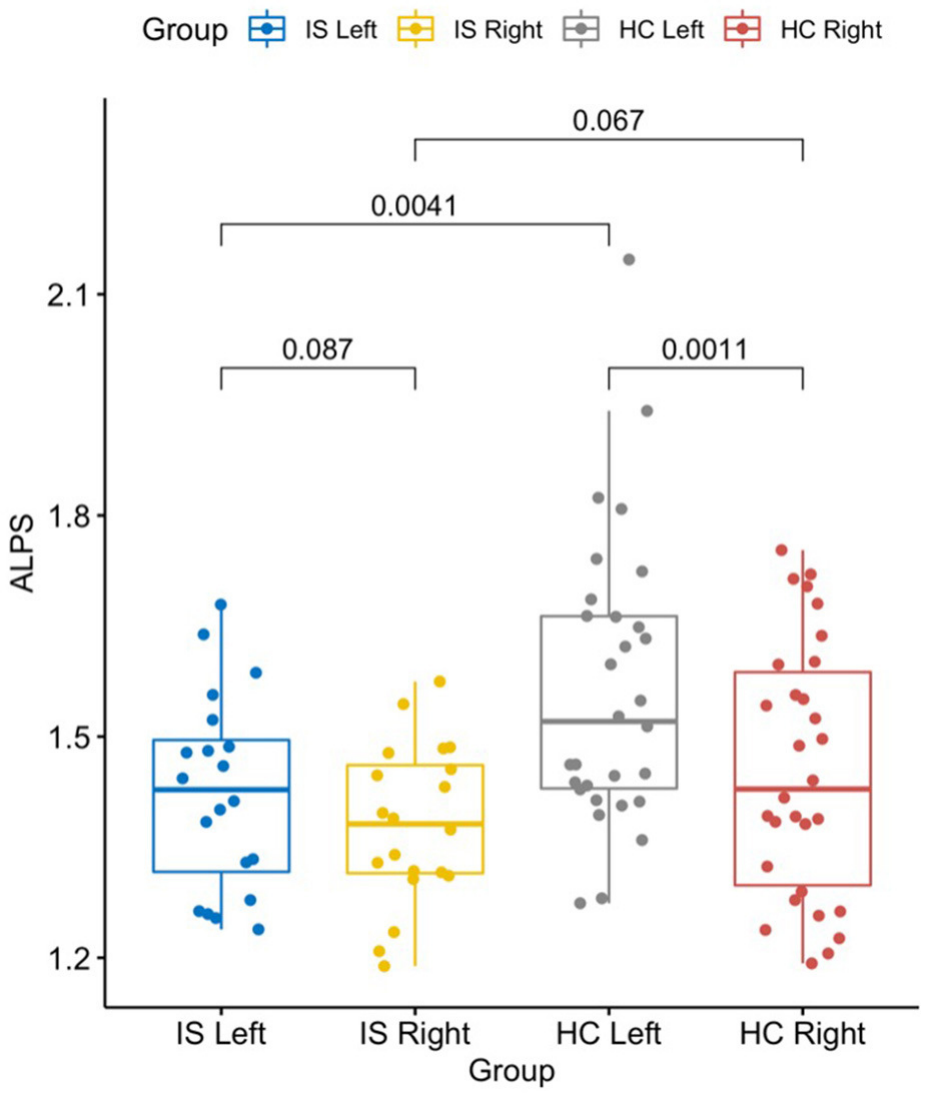
The graph shows a significantly lower DTI-ALPS index in the left/stroke side of the brain in patients with IS than in HCs (*t* = −3.02, *p* = 0.004). The left side was also significantly higher than the right side in HCs (*t* = 3.62, *p* = 0.001). Statistics shown in the graph were *p*-values. DTI-ALPS, diffusion tensor image analysis along the perivascular space; IS, ischemic stroke; HC, healthy controls.

Since there was a significant lateralization effect of DTI-ALPS for the HC group, the left-side DTI-ALPS was higher than that on the right-side DTI-ALPS (*t* = 3.62, *p* = 0.001). However, this effect was not observed in the IS group (*t* = 1.81, *p* = 0.087) (Figure 4).

### 3.3. Associations between the DTI-ALPS index and FMA scores

**Figure 5:**
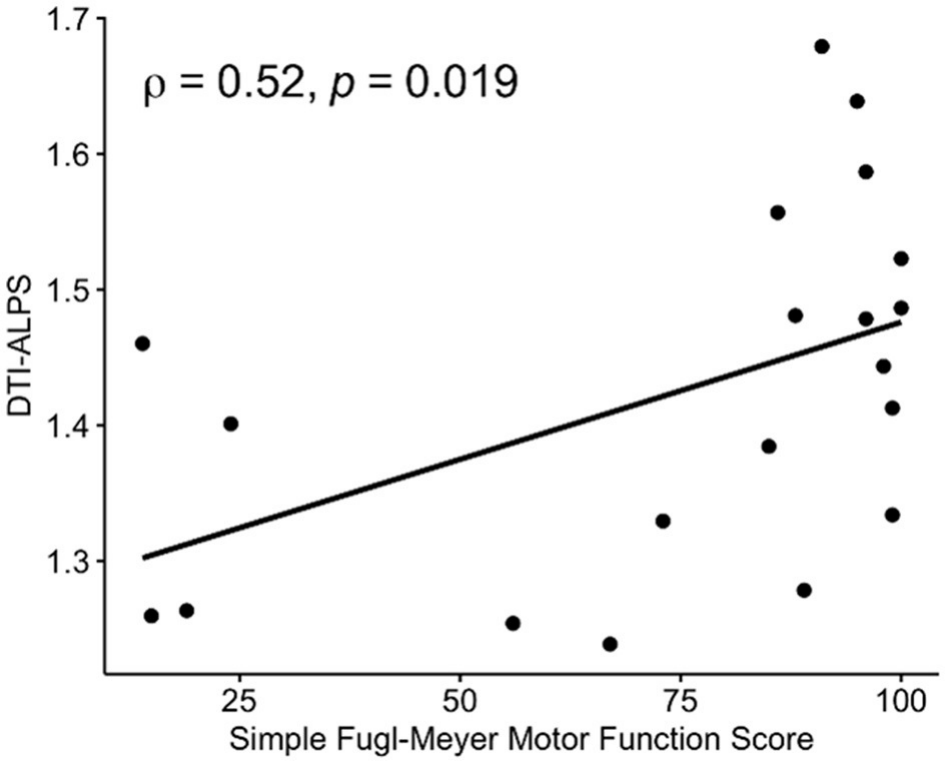
Associations between DTI-ALPS and Fugl-Meyer assessments (FMAs). A significant positive correlation was observed between the DTI-ALPS index and the simple Fugl-Meyer motor function score in patients with IS. DTI-ALPS, diffusion tensor image analysis along the perivascular space.

We found that a higher left DTI-ALPS index was associated with a better simple Fugl-Meyer motor function score (ρ = 0.52, *p* = 0.019, Figure 5) and Fugl-Meyer sensory score (ρ = 0.44, *p* = 0.052) in the IS group. There was no significant association between the right DTI-ALPS index and simple Fugl-Meyer motor function score (ρ = 0.20, *p* = 0.396) and the Fugl-Meyer sensory score (ρ = −0.07, *p* = 0.759).

### 3.4. Comparison of bilateral FA and MD of CST in subacute IS

In the subacute IS group, FA (*t* = −0.36, *p* = 0.721) and MD (*t* = −1.69, *p* = 0.102) on the left were reduced more than those on the right. There was no significant correlation between the bilateral CST metrics and simple Fugl-Meyer motor function score in patients with IS (refer to Supplementary Figure S1).

### 3.5. Associations between the DTI-ALPS index and CST metrics

**Figure 6:**
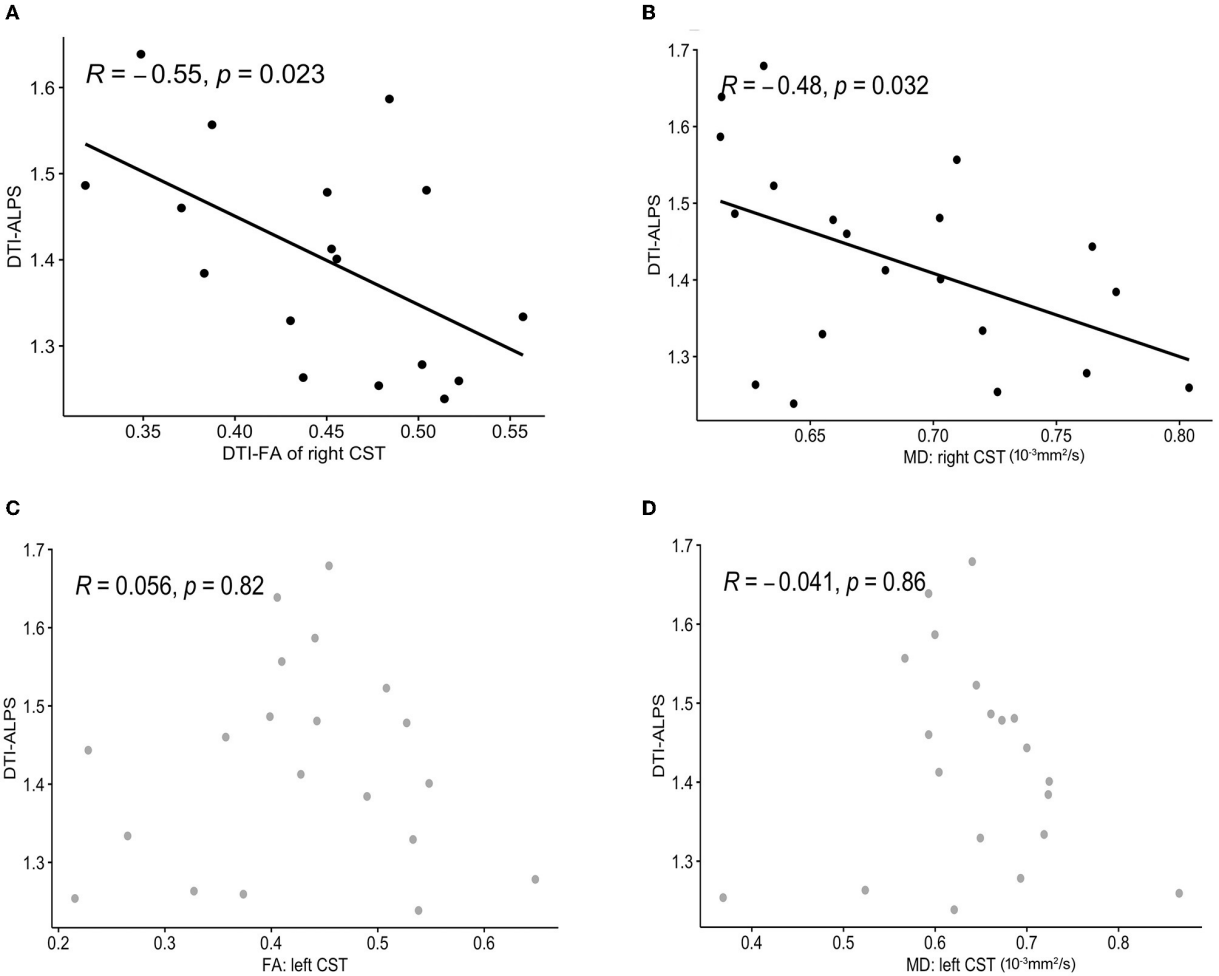
Correlation analysis between the DTI-ALPS index and CST metrics. A significant negative correlation is observed between the left DTI-ALPS index and FA **(A)**, MD **(B)** of the right CST. There was no significant correlation between the left DTI-ALPS index and the left CST: FA **(C)**, MD **(D)**. DTI-ALPS, diffusion tensor image analysis along the perivascular space; CST, corticospinal tract; FA, fractional anisotropy; MD, mean diffusivity.

After controlling for age and time since stroke onset, there were significant negative correlations between the left DTI-ALPS index and the white matter microstructure integrity indices of the right CST: FA (*R* = −0.55, *p* = 0.023) and MD (*R* = −0.48, *p* = 0.032) (Figure 6). There was no significant correlation between the left DTI-ALPS index and the left CST: FA (*R* = 0.056, *p* = 0.82) and MD (*R* = −0.041, *p* = 0.86).

### 3.6. The influence of FA of CC on the DTI-ALPS index

The results showed no significant correlations between the DTI-ALPS index and FA of CC in the IS group (Supplementary Figure S3): the left DTI-ALPS index and FA of the left CC (*R* = 0.13, *p* = 0.57), the right DTI-ALPS index and FA of the right CC (*R* = 0.035, *p* = 0.88), the left DTI-ALPS index and the mean FA of the left CC (*R* = 0.27, *p* = 0.26), and the right DTI-ALPS index and the mean FA of the right CC (*R* = −0.22, *p* = 0.24). In addition, there was also no significant correlation between the DTI-ALPS index and FA of CC in the HC group (refer to Supplementary Figure S4 for the detailed results).

## 4. Discussion

In this study, the IS group had a lower DTI-ALPS value on the affected side of the CST than that of the HC group, reflecting glymphatic dysfunction in patients with IS. In the IS group, a higher DTI-ALPS index was associated with better Fugl-Meyer scores, and there were significant negative associations between the left DTI-ALPS and the right CST (FA and MD). These findings suggest that ALPS can be an MR biomarker of motor dysfunction in patients with subacute stroke and shed light on possible medical interference for IS.

### 4.1. Damaged glymphatic system in stroke patients

Structural changes induced by strokes can significantly alter the characteristics of tissue water diffusion (Muñoz Maniega et al., 2004). The characteristics of subacute IS are blood–brain barrier damage and brain edema (Kanekar et al., 2012). Cerebral edema is a serious complication of IS, and its severity can predict the prognosis of long-term motor function of IS patients (Stokum et al., 2016). Structural damage of the blood–brain barrier and high expression of astrocyte aquaporins following stroke can lead to disruption of lymphatic transport and reduced CSF flow, which can exacerbate brain edema (Randolph et al., 2017; Chen J. et al., 2021; Li et al., 2021) and thus affect motor function outcomes. Animal studies have provided evidence that, in patients with IS, the glymphatic system was seriously damaged and that the clearance of ISF was reduced after IS (Arbel-Ornath et al., 2013; Gaberel et al., 2014; Lin et al., 2020; Ji et al., 2021; Lv et al., 2021). Our results found that patients with subacute IS had lower ALPS indices, similar to the results observed in previous studies (Wang M. et al., 2017; Toh and Siow, 2021b). Stroke is one of the disorders that share the characteristics of dysfunction of the glymphatic system or other mechanisms related to the dynamics of the ISF (Taoka, 2021; Taoka et al., 2022b) and belongs to the CNS interstitial fluidopathy. The new concept proposed by Taoka contributes to the understanding of the pathogenic mechanism of various diseases related to interstitial transport or fluid dynamics (Taoka and Naganawa, 2021). This finding indicated that patients with IS had a damaged glymphatic system, which might be related to the dysfunction of ISF clearance in the glymphatic system, resulting in delaying the subsidence of cerebral edema and affecting the state of motor function. However, the mechanism underlying the impairment of glymphatic function after IS remains unknown. Possible reasons are decreased arterial pulsation, enlarged perivascular space, changes in AQP4 expression and distribution, or swollen astrocytes (Wang et al., 2023). Other physiological glymphatic factors, such as meningeal lymphatic and transvenous efflux rates and the CSF production rate, may also change glymphatic function after IS (Li et al., 2022). More research is needed to look at pathophysiological changes in the brain lymphatic system so as to discover the mechanism and therapeutic target of brain edema after stroke.

### 4.2. Relationship between damaged glymphatic system and motor dysfunction in IS patients

The present study also found that there were significant positive correlations between the DTI-ALPS index and FMA. Previous studies have revealed that the modulation of brain lymphatic activity could affect IS outcomes. AQP4, one of the components of the glymphatic system, is involved in the formation and resolution of edema. In AQP4 knockout mice, brain edema after ischemia was reduced by 35% (Manley et al., 2000). Moreover, acute inhibition of AQP4 with TGN-020 could promote sensorimotor recovery in the subacute stage (Sun et al., 2022). There is evidence that meningeal glymphatic vessels connect with deep cervical lymph nodes (Aspelund et al., 2015; Louveau et al., 2015). Then, in the animal model of focal cerebral ischemia, surgical resection of superficial cervical lymph nodes connected with meningeal glymphatic vessels could block systemic inflammation caused by damaged brain signals, improve post-stroke inflammation, and reduce brain injury (Esposito et al., 2019). All these findings showed that the improvement of glymphatic activity after IS could effectively reduce brain edema and promote recovery from motor dysfunction, which explained the connection between glymphatic activity and motor dysfunction. One of the principles of the treatment of IS is to remove harmful metabolites (Zhu et al., 2022). Recent studies have found that voluntary wheel operation accelerated the clearance of glymphatic function and protected mouse synaptic function (He et al., 2017). Some studies have shown that the treatment *via* the extracellular space of the brain after stroke can reduce cerebral vasospasm (Zhang et al., 2012), vascular permeability (Zhou et al., 2013), and brain edema (Yan et al., 2012), which was beneficial for improving neurological function (including motor sensory function) (Xu et al., 2011). This research provides an association between glymphatic dysfunction and IS, which may provide a theoretical basis for the development of new clinical therapeutics. Our finding demonstrated that lymphatic function was abnormal during the period of subacute IS, which is consistent with prior studies. Extensive reactive astrogliosis occurred 14 days following diffuse IS; during this time, global lymphatic function returned to normal as AQP4 expression returned to the baseline levels (Wang et al., 2012), while focal glymphatic impairment might persist for a long time (Wang M. et al., 2017). Related studies have shown that the glymphatic injury persisted 28 days after the brain injury (Iliff et al., 2014). These findings indicated that changes in glymphatic activity and motor dysfunction after IS may be related to time since stroke onset, which needs to be confirmed by longitudinal studies.

### 4.3. Association between damaged glymphatic system and CST in stroke patients

In this study, there were significant negative correlations between the left DTI-ALPS index and the right CST (FA and MD). We found that the integrity of CST on the affected side was impaired after stroke, which was consistent with a previous study (Peters et al., 2021). Longitudinal studies showed that FA in infarcts decreased gradually from the acute to the subacute phase, while MD initially decreased and then increased (Kern et al., 2022). Decreased FA might be related CST axonal damage and degeneration (Chang et al., 2017), and increased MD might be associated with vasogenic edema after stroke (Kern et al., 2022; Lee S.-Y. et al., 2022), which has a great impact on motor dysfunction after stroke.

Diffusion tensor image metrics can be used as an imaging evaluation index for monitoring motor function impairment (Yang et al., 2022) or motor recovery after stroke (Puig et al., 2017). Findlater et al. (2019) found that low CST-FA was associated with poor motor performance 1 month after stroke. Research on hypoxic-ischemia-induced stroke thrombosis model in adult mice and chronic stroke patients showed that the FA values of bilateral CSTs decreased after stroke due to the loss of axonal or CST integrity (Yu et al., 2009; Shereen et al., 2011; Lakhani et al., 2017). Most studies have reported axonal remodeling during spontaneous recovery after stroke (Wahl et al., 2014, 2017). Growing evidence suggested that CST axonal remodeling in the contralateral motor system also contributed to spontaneous motor recovery after stroke (Schaechter et al., 2009; Yeo and Jang, 2012; Okabe et al., 2017). Liu et al. (2008) used CST tracing to investigate contralateral neuronal reorganization after IS and administration in adult rats and showed that the treatment to improve neurological outcomes could also enhance neuronal remodeling in the contralateral intact hemisphere through CST axonal remodeling, which might be a benefit for motor recovery. Another study on a rodent stroke model clarified the role of brain astrocytes in functional compensation following IS and showed that the activation of contralateral astrocytes might be involved in the functional recovery of the contralateral region of the lesion by clearing extracellular glutamate (Takatsuru et al., 2013). The integrity of myelin plays an important role in maintaining ISF drainage in adults. In areas affected by stroke, remyelination is important in the process of functional recovery (Park et al., 2022). Intact myelin can serve as a barrier structure for ISF drainage, which is beneficial for the development of cognitive and motor or sensory abilities of the brain (Wang et al., 2021). This may explain why the association between the ipsilateral DTI-ALPS index and the contralateral CST in our study may be related to CST axonal remodeling and the clearance function of the glymphatic system.

### 4.4. Lateralization of DTI-ALPS

In contrast to the result of a recent study (Zhang et al., 2022), the DTI-ALPS value on the left side is higher than that on the right side in our HC participants. Given the small sample sizes of both studies and the difference in age distributions, it was difficult to say whether there should be a lateralization effect on human brain glymphatic systems. A reverse trend between brain lymphatic system function and human age has currently been confirmed (Jessen et al., 2015; Lee D. A. et al., 2022). In addition, handedness and the dominant brain side influence the thickness of the superior longitudinal fasciculus (Chormai et al., 2022). This may have an impact on the measurement of ALPS, and therefore, the results may not be as accurate as expected, which also contributes to laterality. The current demonstration of laterality needs to be treated with caution, and further investigation is needed to clarify the existence and causes of laterality in the brain lymphatic system.

To the best of our knowledge, this is the first study to investigate whether the ALPS index is affected by commissural fibers traveling in the left and right directions. There was no significant correlation between the FA values of the CC and the ALPS index in both the IS and HC groups. Based on the abovementioned preliminary results, we suggest that the ALPS index is an appropriate measurement in the present study and can reflect the state of the glymphatic system. Nevertheless, this preliminary conclusion needs to be further confirmed by more rigorous studies of large samples.

This study has some limitations. First, the small sample size of this study requires prudence in interpreting the results. Second, although the onset time of IS was 7–40 days, most of them were concentrated within 20 days, and glymphatic function may change over time, which may have some impact on the findings (Toh and Siow, 2021b). Third, because the DSI acquisition method uses multiple *b*-value acquisitions, it may not be the optimal DTI-ALPS scanning protocol. According to a recent study (Taoka et al., 2022a), there was a high correlation between the DTI-ALPS value obtained using different scanning parameters, indicating that our study protocol is also suitable for this type of research. Finally, the patient's prognosis for motor function could not be determined from our findings on the relationship between the DTI-ALPS value and motor function due to the cross-sectional study design.

## 5. Conclusion

In conclusion, a decreased DTI-ALPS index indicates glymphatic system dysfunction in patients with subacute IS and may serve as an MR biomarker for motor dysfunction in these patients. These findings could help us understand the pathophysiological mechanisms of IS and develop alternative treatment options for post-IS glymphatic activity.

## Data availability statement

The raw data supporting the conclusions of this article will be made available by the authors, without undue reservation.

## Ethics statement

The studies involving human participants were reviewed and approved by the Medical Research Ethics Committee of Xi'an Daxing Hospital. The patients/participants provided their written informed consent to participate in this study.

## Author contributions

YQ: conceptualization, investigation, and writing—review and editing. XinL: writing—original draft. YaQ: investigation and data curation. HZ: data curation. YiQ: software and visualization. XiaL, YZ, and WH: investigation. LW: conceptualization, methodology, software, and writing—review and editing. MZ: conceptualization, supervision, and writing—review and editing. All authors contributed to the article and approved the submitted version.
