# Supplementary material for: DTI-ALPS: An MR biomarker for motor dysfunction in patients with subacute ischemic stroke
Source: Front Neurosci. 2023 Mar 31;17:1132393. doi: 10.3389/fnins.2023.1132393 (PMC10102345; doi:10.3389/fnins.2023.1132393)
Supplement: Supplementary file 1 [file Data_Sheet_1.docx]

***Supplemental Material***

**DTI-ALPS: An MR biomarker for motor dysfunction in patients with subacute** **ischaemic stroke**

Yue Qin^1,^^2†^, Xin Li^2†^, Yanqiang Qiao^1,2^^†^, Huili Zou^3^, Yifan Qian^2^, Xiaoshi Li^2^, Yinhu Zhu^2^, Wenli Huo^1^, Lei Wang^1,2*^, Ming Zhang^1*^

^1^ Department of Medical Imaging, The First Affiliated Hospital of Xi'an Jiaotong University, Xi'an, China.

^2^ Department of Radiology, Xi’an Daxing Hospital, Xi’an, China.

^3^ Department of Rehabilitation Medicine, Xi’an Daxing Hospital, Xi’an, China.

^*^ **Correspondence:** Ming Zhang: zmdx_2022@163.com; Lei Wang: shagojo@163.com.

^†^ These authors contributed equally to this work.

**1 Supplementary Table**

Table S1 The b table of one patient’s DSI data.

| Volume Index | b value | bx | by | bz |
| --- | --- | --- | --- | --- |
| 1 | 0 | 0 | 0 | 0 |
| 2 | 200 | -0.0060722 | -0.953734 | 0.300591 |
| 3 | 200 | 0.00761994 | -0.305914 | -0.952029 |
| 4 | 200 | 0.999995 | -0.0017328 | -0.0027001 |
| 5 | 350 | -0.713223 | -0.670125 | 0.205536 |
| 6 | 400 | -0.697921 | -0.214977 | -0.683148 |
| 7 | 400 | 0.00109097 | -0.888267 | -0.459326 |
| 8 | 400 | 0.00972105 | 0.448745 | -0.893607 |
| 9 | 350 | 0.703709 | -0.675974 | 0.218752 |
| 10 | 400 | 0.707825 | -0.220167 | -0.671201 |
| 11 | 550 | -0.575399 | -0.7239 | -0.380637 |
| 12 | 550 | -0.566312 | 0.369039 | -0.736954 |
| 13 | 550 | 0.576503 | -0.728347 | -0.370345 |
| 14 | 550 | 0.581556 | 0.365246 | -0.726903 |
| 15 | 750 | -0.0061032 | -0.952506 | 0.304459 |
| 16 | 750 | 0.00760416 | -0.308366 | -0.951237 |
| 17 | 750 | 0.999994 | -0.0030284 | 0.00196039 |
| 18 | 950 | -0.897928 | -0.422065 | 0.124846 |
| 19 | 950 | -0.888651 | -0.135069 | -0.438241 |
| 20 | 950 | -0.453407 | -0.84995 | 0.268343 |
| 21 | 950 | -0.437921 | -0.274633 | -0.856038 |
| 22 | 950 | -0.0020561 | -0.988268 | -0.152713 |
| 23 | 950 | 0.00407228 | -0.700089 | -0.714044 |
| 24 | 950 | 0.00953688 | 0.145961 | -0.989244 |
| 25 | 950 | 0.00891569 | 0.707953 | -0.706203 |
| 26 | 950 | 0.442106 | -0.853258 | 0.276573 |
| 27 | 950 | 0.451168 | -0.277794 | -0.848103 |
| 28 | 950 | 0.892532 | -0.428315 | 0.141185 |
| 29 | 950 | 0.895536 | -0.141297 | -0.42196 |
| 30 | 1150 | -0.814724 | -0.511129 | -0.273808 |
| 31 | 1150 | -0.808399 | 0.263437 | -0.526395 |
| 32 | 1150 | -0.409859 | -0.900831 | -0.143245 |
| 33 | 1150 | -0.402944 | -0.638273 | -0.655931 |
| 34 | 1150 | -0.397295 | 0.134804 | -0.907736 |
| 35 | 1150 | -0.39888 | 0.648092 | -0.648746 |
| 36 | 1150 | 0.405804 | -0.903812 | -0.135822 |
| 37 | 1150 | 0.410082 | -0.641204 | -0.648606 |
| 38 | 1150 | 0.414428 | 0.13199 | -0.90046 |
| 39 | 1150 | 0.414869 | 0.645346 | -0.641414 |
| 40 | 1150 | 0.815998 | -0.516822 | -0.258927 |
| 41 | 1150 | 0.81969 | 0.257754 | -0.511538 |
| 42 | 1500 | -0.712085 | -0.670384 | 0.208615 |
| 43 | 1500 | -0.699332 | -0.216303 | -0.681284 |
| 44 | 1500 | 0.00105582 | -0.889924 | -0.456108 |
| 45 | 1500 | 0.00971946 | 0.450185 | -0.892883 |
| 46 | 1500 | 0.703462 | -0.675325 | 0.221533 |
| 47 | 1500 | 0.710122 | -0.221223 | -0.668422 |
| 48 | 1700 | -0.667809 | -0.734569 | -0.120167 |
| 49 | 1700 | -0.661905 | -0.520418 | -0.539488 |
| 50 | 1700 | -0.657259 | 0.111576 | -0.74536 |
| 51 | 1700 | -0.6584 | 0.53104 | -0.533391 |
| 52 | 1700 | -0.331575 | -0.838118 | -0.433148 |
| 53 | 1700 | -0.322972 | 0.425876 | -0.845174 |
| 54 | 1700 | -0.0061225 | -0.951731 | 0.306871 |
| 55 | 1700 | 0.00759521 | -0.309751 | -0.950787 |
| 56 | 1700 | 0.333391 | -0.840493 | -0.427108 |
| 57 | 1700 | 0.341133 | 0.423586 | -0.839168 |
| 58 | 1700 | 0.664747 | -0.73922 | -0.108006 |
| 59 | 1700 | 0.667991 | -0.52506 | -0.527351 |
| 60 | 1700 | 0.671515 | 0.106938 | -0.733234 |
| 61 | 1700 | 0.671716 | 0.526397 | -0.521253 |
| 62 | 1700 | 0.999982 | -0.0036536 | 0.00481298 |
| 63 | 1850 | -0.950978 | -0.297197 | 0.0855318 |
| 64 | 1900 | -0.944986 | -0.0950068 | -0.313011 |
| 65 | 1850 | -0.322575 | -0.901592 | 0.28823 |
| 66 | 1900 | -0.307899 | -0.292816 | -0.905239 |
| 67 | 1900 | -0.0034002 | -0.999945 | -0.0098856 |
| 68 | 1900 | 0.00527517 | -0.593257 | -0.804996 |
| 69 | 1900 | 0.00914416 | 0.00548366 | -0.999943 |
| 70 | 1900 | 0.00825674 | 0.801215 | -0.59832 |
| 71 | 1850 | 0.310807 | -0.903852 | 0.294026 |
| 72 | 1900 | 0.322167 | -0.295031 | -0.899536 |
| 73 | 1850 | 0.947109 | -0.303967 | 0.102898 |
| 74 | 1900 | 0.949804 | -0.101668 | -0.295866 |
| 75 | 2050 | -0.903351 | -0.376717 | -0.20504 |
| 76 | 2050 | -0.898907 | 0.195685 | -0.392014 |
| 77 | 2050 | -0.304883 | -0.952312 | -0.0121823 |
| 78 | 2100 | -0.295699 | -0.564786 | -0.770441 |
| 79 | 2100 | -0.291694 | 0.00624692 | -0.956491 |
| 80 | 2050 | -0.292893 | 0.765157 | -0.573366 |
| 81 | 2050 | 0.298266 | -0.954459 | -0.0066787 |
| 82 | 2100 | 0.305628 | -0.56691 | -0.764987 |
| 83 | 2100 | 0.309006 | 0.00415033 | -0.951051 |
| 84 | 2050 | 0.30851 | 0.763091 | -0.567903 |
| 85 | 2050 | 0.904211 | -0.383177 | -0.188623 |
| 86 | 2050 | 0.907176 | 0.189456 | -0.375683 |
| 87 | 2250 | -0.575602 | -0.725104 | -0.378029 |
| 88 | 2250 | -0.567844 | 0.37015 | -0.735216 |
| 89 | 2250 | 0.57733 | -0.729128 | -0.367508 |
| 90 | 2250 | 0.583737 | 0.36613 | -0.724707 |
| 91 | 2450 | -0.83593 | -0.524627 | 0.161208 |
| 92 | 2450 | -0.826168 | -0.16921 | -0.537415 |
| 93 | 2450 | -0.56021 | -0.789692 | 0.250104 |
| 94 | 2450 | -0.546726 | -0.255987 | -0.797221 |
| 95 | 2450 | -0.0008756 | -0.962262 | -0.272123 |
| 96 | 2450 | 0.00292773 | -0.784021 | -0.620727 |
| 97 | 2450 | 0.0097263 | 0.267529 | -0.963501 |
| 98 | 2450 | 0.00933667 | 0.616456 | -0.787334 |
| 99 | 2450 | 0.550026 | -0.793567 | 0.260236 |
| 100 | 2450 | 0.559383 | -0.259848 | -0.787127 |
| 101 | 2450 | 0.82906 | -0.530589 | 0.17645 |
| 102 | 2450 | 0.834532 | -0.175055 | -0.52241 |
| 103 | 2650 | -0.802736 | -0.588103 | -0.0987359 |
| 104 | 2650 | -0.798097 | -0.416715 | -0.435189 |
| 105 | 2650 | -0.794348 | 0.0904803 | -0.600686 |
| 106 | 2650 | -0.795239 | 0.426937 | -0.430489 |
| 107 | 2650 | -0.53728 | -0.843299 | -0.01331 |
| 108 | 2650 | -0.528773 | -0.499972 | -0.685877 |
| 109 | 2650 | -0.525198 | 0.00660209 | -0.850955 |
| 110 | 2650 | -0.526598 | 0.679498 | -0.510859 |
| 111 | 2650 | -0.267837 | -0.926411 | -0.264626 |
| 112 | 2650 | -0.263919 | -0.754669 | -0.600685 |
| 113 | 2650 | -0.257108 | 0.2588 | -0.931084 |
| 114 | 2650 | -0.257409 | 0.595127 | -0.761292 |
| 115 | 2650 | 0.266054 | -0.928299 | -0.259761 |
| 116 | 2650 | 0.269468 | -0.756551 | -0.595833 |
| 117 | 2650 | 0.275764 | 0.256947 | -0.926247 |
| 118 | 2650 | 0.275314 | 0.593283 | -0.75645 |
| 119 | 2650 | 0.531533 | -0.84703 | -0.0035565 |
| 120 | 2650 | 0.537699 | -0.503695 | -0.676145 |
| 121 | 2650 | 0.540673 | 0.00288151 | -0.841228 |
| 122 | 2650 | 0.540564 | 0.675773 | -0.501121 |
| 123 | 2650 | 0.80017 | -0.593843 | -0.0841309 |
| 124 | 2650 | 0.802869 | -0.422405 | -0.420683 |
| 125 | 2650 | 0.805683 | 0.0849166 | -0.586229 |
| 126 | 2650 | 0.805815 | 0.421451 | -0.415981 |
| 127 | 3000 | -0.0061288 | -0.95148 | 0.307651 |
| 128 | 3000 | 0.0075941 | -0.309923 | -0.950731 |
| 129 | 3000 | 0.999978 | -0.0035762 | 0.00564621 |

Table S2 The DTI-ALPS index and CST metrics of ischaemic stroke and healthy control.

|  | Ischaemic stroke | | | | Healthy control | | | |
| --- | --- | --- | --- | --- | --- | --- | --- | --- |
|  | Left | Right | *t* | *P* | Left | Right | *t* | *P* |
| DTI-ALPS | 1.424±0.132 | 1.381±0.172 | 1.81 | 0.087 | 1.565±0.197 | 1.454±0.107 | 3.62 | 0.001 |
| FA | 0.427±0.112 | 0.438±0.076 | -0.36 | 0.721 | 0.499±0.089 | 0.458±0.086 | 1.79 | 0.079 |
| MD | 0.643±0.098 | 0.686±0.059 | -1.69 | 0.102 | 0.634±0.031 | 0.643±0.028 | -1.21 | 0.232 |
| AD | 0.977±0.196 | 1.051±0.132 | -1.41 | 0.168 | 1.020±0.081 | 1.000±0.094 | 0.90 | 0.374 |
| RD | 0.475±0.080 | 0.503±0.050 | -1.29 | 0.204 | 0.440±0.055 | 0.464±0.049 | -1.78 | 0.081 |

*Abbreviations:*

*DTI-ALPS, diffusion tensor image analysis along the perivascular space; CST, corticospinal tract; FA, fractional anisotropy; MD, mean diffusivity; AD, axial diffusivity; RD, radial diffusivity. The MD, AD, and RD values have a unit of* *10^-3^ mm^2^/s.*

**2 Supplementary Figures**


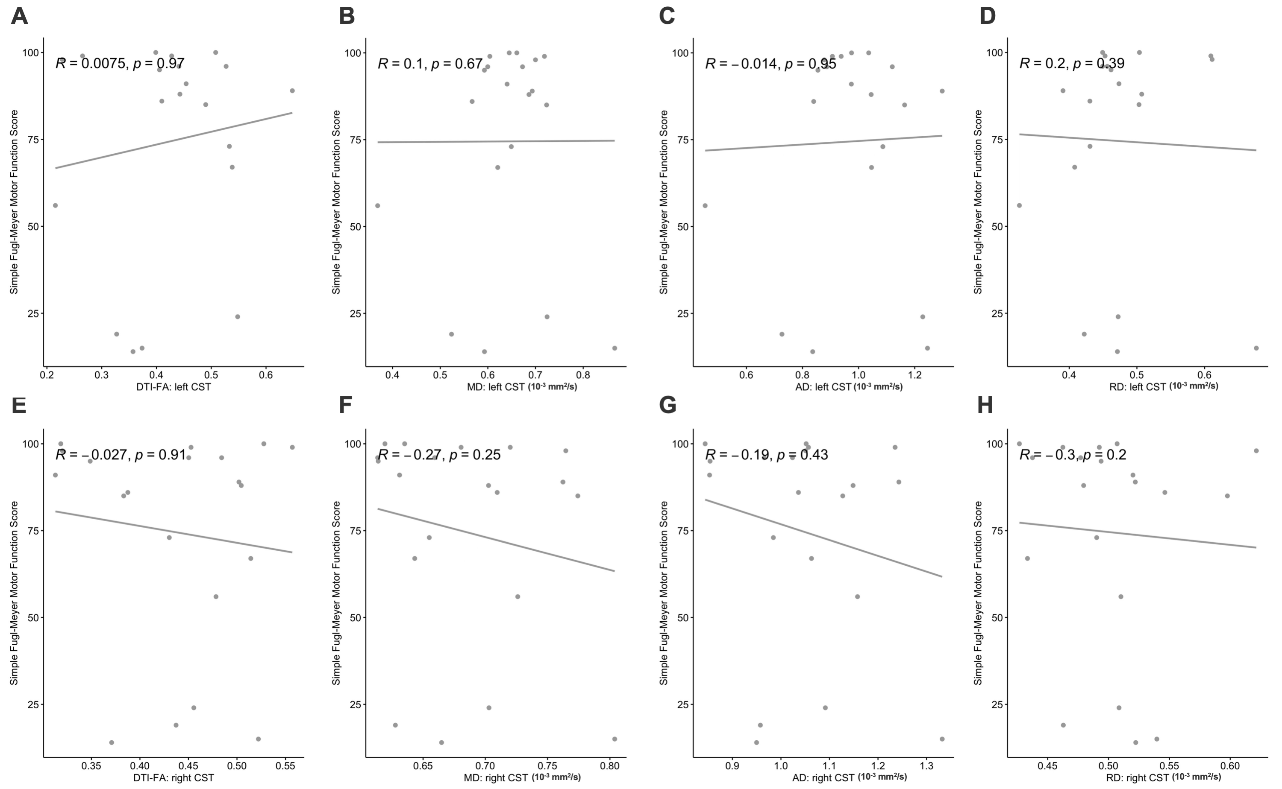


Fig. S1 Associations between CST metrics and Fugl-Meyer assessments. There was no significant correlation between the bilateral CST metrics (FA, MD, AD, RD) and simple Fugl-Meyer motor function score in patients with ischemic stroke.

*Abbreviations:*

*DTI-ALPS, diffusion tensor image analysis along the perivascular space; CST, corticospinal tract; FA, fractional anisotropy; MD, mean diffusivity; AD, axial diffusivity; RD, radial diffusivity.*


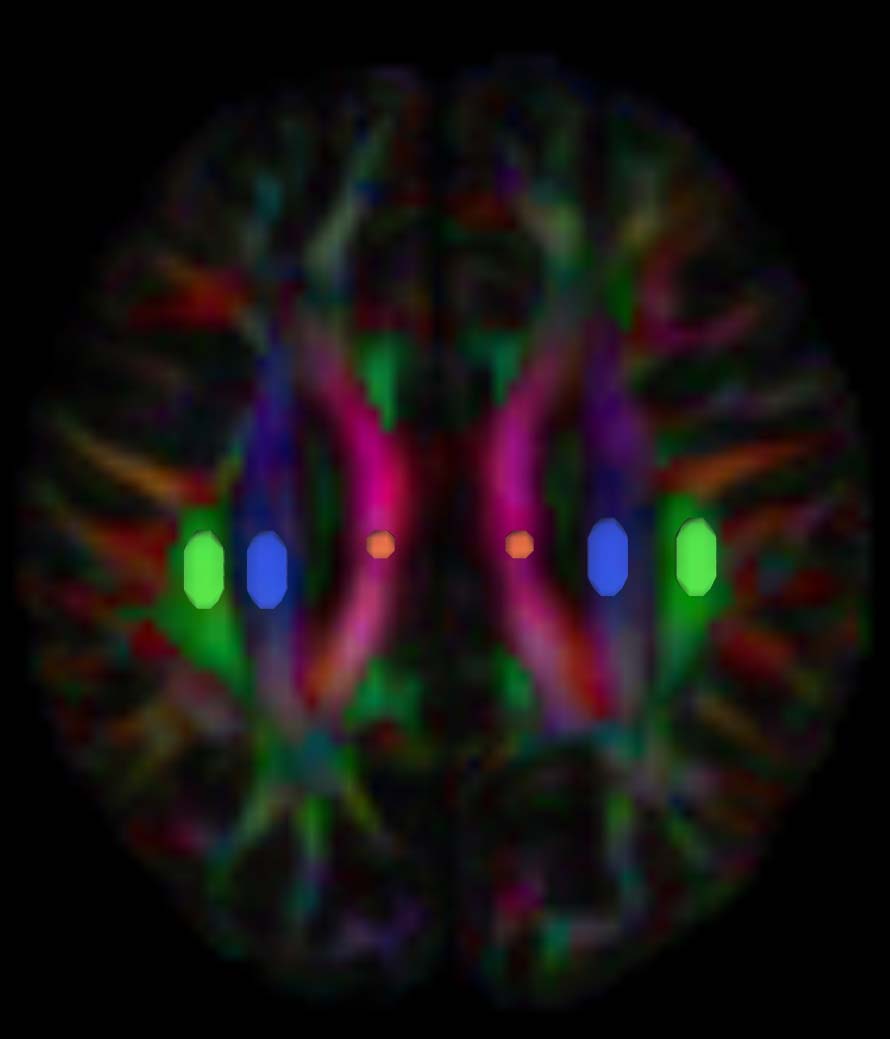


Fig. S2 The DTI color map shows the direction of the projection fibers (z-axis, blue), association fibers (y-axis, green), and subcortical fibers (x-axis, red). Three regions of interest (ROIs) are placed to measure diffusivities of the commissural fibers (projection area), association (association area) fibers, and commissural (corpus callosum) fibers.


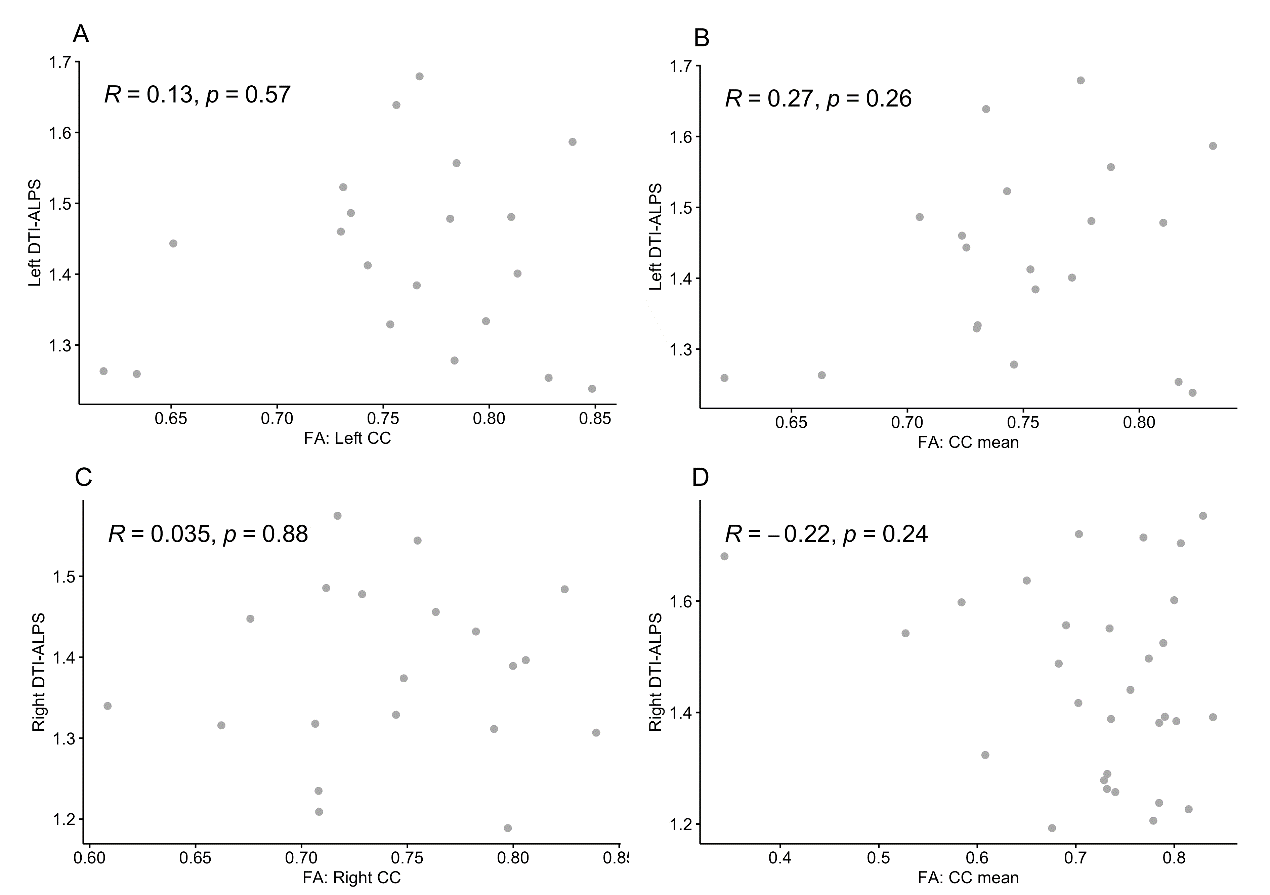


Fig. S3 There was no significant correlation between the FA values of the corpus callosum and the ALPS index in the ischaemic stroke group.


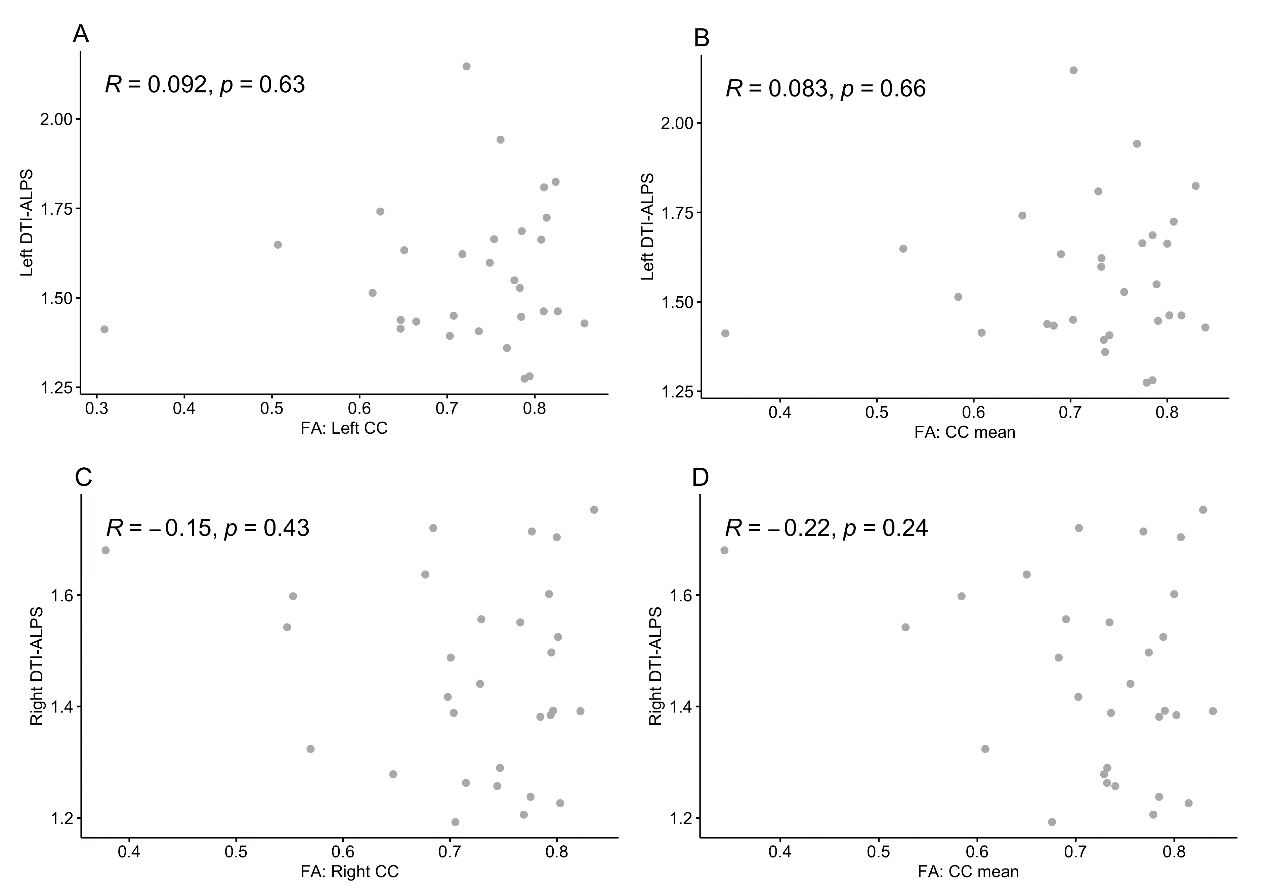


Fig. S4 There was no significant correlation between the FA values of the corpus callosum and the ALPS index in the healthy control group.

*Abbreviations:*

*DTI-ALPS, diffusion tensor image analysis along the perivascular space; DTI, diffusion tensor image; FA, fractional anisotropy; CC, corpus callosum.*

**3 Supplementary Analysis**

**Correlation analysis of** **infarct volume and ALPS index**

The 3D Slicer software was used to outline the stroke lesion layer by layer in the FLAIR image to obtain the infarct volume. The correlation of infarct volume and ALPS index was evaluated by the Pearson correlation analysis. Correlation analysis resulted in a non-significant correlation between infarct volume and bilateral ALPS index (left: *R* = -0.24, *P* = 0.31; right: *R* = -0.05, *P* = 0.84).
